# LncRNA AP003469.2 promotes hepatocellular carcinoma progression by regulating cuproptosis via the miR-4732-3p/LIPT1 axis

**DOI:** 10.3389/fonc.2026.1874176

**Published:** 2026-10-01

**Authors:** Jinlong Huo, Shuman Kuang, Lin Wu, Dong Ou

**Affiliations:** 1 Department of Breast and Thyroid Surgery, The Third Affiliated Hospital of Zunyi Medical University (The First People’s Hospital of Zunyi), Zunyi, Guizhou, China; 2 Department of General Surgery, Digestive Disease Hospital, Affiliated Hospital of Zunyi Medical University, Zunyi, Guizhou, China; 3 Guizhou Provincial Key Laboratory of Digestive System Diseases, Zunyi, Guizhou, China; 4 Department of Oncology, The Third Affiliated Hospital of Zunyi Medical University (The First People’s Hospital of Zunyi), Zunyi, Guizhou, China

**Keywords:** cuproptosis, hepatocellular carcinoma, LIPT1, lncRNA AP003469.2, miR-4732-3p

## Abstract

**Background:**

Hepatocellular carcinoma (HCC) is a malignant tumor characterized by high recurrence and metastasis rates, as well as a poor prognosis. Cuproptosis is a form of programmed cell death driven by abnormal copper-ion metabolism. Existing studies have shown that its occurrence is closely regulated and associated with the progression of HCC. However, it is still unclear how long non-coding RNAs (lncRNAs) control the process in HCC. The purpose of this work is to clarify how the lncRNA AP003469.2 influences cuproptosis and promotes the development of HCC.

**Methods:**

This study utilized RNA sequencing data from the TCGA-LIHC dataset to screen for cuproptosis-related lncRNAs exhibiting significant expression differences. Following the initial screening, the expression of lncRNA AP003469.2 was validated using qRT-PCR. Subsequent *in vitro* and *in vivo* studies confirmed AP003469.2’s biological role in HCC. To understand the fundamental mechanisms, we determined the subcellular localization of the proteins using nuclear–cytoplasmic fractionation and fluorescence *in situ* hybridization (FISH). The regulatory relationships between miR-4732-3p and LIPT1 and between AP003469.2 and miR-4732-3p were then verified using dual-luciferase reporter assays. In addition, we utilized Western blotting, copper assays, CCK-8, and immunohistochemistry to evaluate related proteins and markers indicative of cuproptosis.

**Results:**

A poor prognosis was observed to be positively correlated with elevated expression of AP003469.2 in HCC. In both *in vivo* and *in vitro* experimental methods, AP003469.2 stimulated important malignant characteristics in HCC cells, including invasion, migration, and proliferation. Furthermore, by controlling LIPT1 expression and modulating copper homeostasis and important proteins, including FDX1, SLC31A1, and HSP70, AP003469.2 acts as a molecular sponge for miR-4732-3p, attenuating cuproptosis.

**Conclusion:**

Our research demonstrated that the AP003469.2/miR-4732-3p/LIPT1 axis could suppress cuproptosis to promote HCC progression. Therefore, AP003469.2 may serve as a target for HCC treatment or diagnosis.

## Background

Hepatocellular carcinoma (HCC) is the fifth most prevalent cancer and the third most common cause of cancer-related mortality worldwide (1–3). The five-year survival rate for those with HCC is still less than 20%, despite tremendous advancements in both diagnostic and therapeutic approaches (4–6). The substantial rates of recurrence and metastasis are significant factors contributing to the unfavorable prognosis for HCC, and these continue to be major obstacles in clinical practice. Consequently, clarifying the molecular pathways that drive HCC progression and discovering novel therapeutic interventions is imperative.

As an essential micronutrient, copper fulfills critical functions in key biological processes, including mitochondrial respiration, managing oxidative stress, and catalyzing enzymatic reactions (7–9). Dysregulated copper homeostasis can be cytotoxic, ultimately resulting in a recently discovered mechanism of regulated cell death called cuproptosis (10). Unlike other forms of cell death, the fundamental mechanism of cuproptosis is that copper directly binds to lipoylated proteins in the tricarboxylic acid cycle. This process leads to the instability of iron-sulfur cluster proteins, abnormal protein aggregation, and proteotoxic stress, ultimately triggering cell death (11, 12). Important genes that affect protein lipoylation and copper binding affinity, such as ferredoxin 1 (FDX1) and lipoyltransferase 1 (LIPT1), carefully control this process (13–15). There are other disorders, especially cancers, that have been linked to this unique procedure (16–19).

Research on cuproptosis in HCC is increasingly viewed as a promising avenue for both therapeutic development and diagnostic innovation. The use of cuproptosis-related genes in LASSO-Cox models has shown great promise for precise patient survival outcome prediction (20). Mechanistic studies have demonstrated that dihydrolipoamide S-acetyltransferase (DLAT) facilitates cuproptosis, whereas Elesclomol functions as a cuproptosis inducer in HCC cells, thereby underscoring the critical importance of copper homeostasis in disease progression (21). Furthermore, integrative models combining cuproptosis and ferroptosis demonstrate strong associations with immune microenvironment variables, suggesting that cuproptosis influences immune responses and treatment sensitivity (22). Additionally, p53 has been identified as a cuproptosis promoter operating through the ferredoxin reductase/ferredoxin 1 (FDXR/FDX1) axis, with associated long non-coding RNAs (lncRNAs) and N6-methyladenosine (m^6^A) modifications further expanding the regulatory landscape (23, 24). However, despite these significant advances in understanding copper metabolism and genetic regulation, the systematic characterization of gene expression patterns related to cuproptosis, their prognostic value, and particularly the upstream lncRNA-mediated regulation in HCC remains insufficient.

Long non-coding RNAs, characterized by a length of over 200 nucleotides, are incapable of directly encoding proteins. They provide critical functions at several stages, including the regulation of the chromatin state before transcription, modulation of the transcription process itself, and control of mRNA splicing, translation, and destruction subsequent to transcription (25–27). By altering proteins, signaling pathways, and cellular functions, they contribute significantly to cancer. They often function as competitive endogenous RNAs (ceRNAs) that derepress target mRNAs and sponge microRNAs (miRNAs) (28–31). Among various regulatory mechanisms, recent attention has focused on lncRNA involvement in metal-dependent programmed cell death pathways, including ferroptosis and cuproptosis (32–34). Several lncRNAs have become key regulators of these processes, with PVT1 being a particularly well-characterized example. In ferroptosis regulation, PVT1 promotes cell death by sponging miR-214, which upregulates transferrin receptor 1 (TfR1) expression and modifies intracellular iron pools (35). Beyond ferroptosis, PVT1 also shows functional versatility in cuproptosis; it enhances the cell death pathway in colorectal cancer by epigenetically activating FDX1, thereby increasing tumor sensitivity to cuproptosis (34). Similarly, in head and neck squamous cell carcinoma, the cuproptosis-associated lncRNA JPX promotes malignant behavior. It acts as a competitive endogenous RNA for miR-193b-3p, which relieves miR-193b-3p-mediated repression of the oncogene PLAU and upregulates PLAU expression (36). Despite these mechanistic insights into lncRNA regulation of cuproptosis in various cancers, the specific pathways by which lncRNAs modulate cuproptosis in HCC remain largely unexplored.

Based on these foundational findings, we used integrative bioinformatics approaches combined with experimental validation to identify novel cuproptosis-related lncRNAs in HCC. Systematic analysis revealed lncRNA AP003469.2 as the most significantly upregulated cuproptosis-related lncRNA in HCC, with strong prognostic and diagnostic relevance. In this study, evidence indicates that AP003469.2 accelerates HCC progression by sequestering miR-4732-3p as a competing endogenous RNA. This interaction upregulates LIPT1, which maintains copper homeostasis, reduces proteotoxic stress, and decreases sensitivity to cuproptosis. Our findings identify a novel AP003469.2/miR-4732-3p/LIPT1 regulatory axis and indicate its translational promise for boosting the efficacy of cuproptosis-based treatments in HCC.

## Materials and methods

### Patient specimens

Between July 2022 and December 2023, twenty matched pairs of primary HCC and adjacent non-tumorous tissues were collected from surgical patients at the Department of Hepatobiliary Surgery, Affiliated Hospital of Zunyi Medical University. All surgical patients had not received chemotherapy, radiotherapy, or targeted therapy for HCC before. Following excision, all tissues underwent histological verification, were promptly frozen in liquid nitrogen, and were maintained at -80 °C to facilitate the extraction of proteins and RNA. The Institutional Ethics Committee approved the research (Approval No. ZYFY-2024-1-085), and each subject provided written informed consent.

### Bioinformatics analysis

Data on RNA sequencing (FPKM values) and related clinical details for HCC were sourced from the TCGA-LIHC dataset, which includes 374 tumor samples and 50 nearby non-tumor samples. Thirteen genes linked to cuproptosis are identified using previously published research (10). The “limma” R package was used to perform expression analysis on cuproptosis genes in HCC (with fold change ≥ 0 and FDR ≤ 0.05). Subsequently, univariate Cox regression analysis was employed to identify cuproptosis-related genes associated with the prognosis of HCC patients. Using Pearson correlation analysis, lncRNAs linked to cuproptosis were identified based on prognostic cuproptosis genes, with criteria of |r| exceeding 0.4 and *p*-values under 0.05. Differentially expressed lncRNAs (fold change ≥ 1.0 and FDR ≤ 0.05) were further filtered and intersected with cuproptosis-related lncRNAs to obtain candidate regulators. AP003469.2 showed the most notable dysregulation and was chosen for further study. The receiver operating characteristic (ROC) analysis and the Kaplan-Meier survival curves were used to evaluate the diagnostic and prognostic efficiency, respectively.

Furthermore, clinical information and miRNA expression patterns for HCC patients were extracted from TCGA-LIHC and normalized. The “limma” R package was also used to do expression analysis, using the condition of |log_2_FC| > 0.5 and FDR < 0.05. Prognostic relevance was assessed by multivariate Cox regression, and miRNAs with significant associations (*p* < 0.05) and hazard ratios (HR) < 1 were defined as protective factors. The overlap between these protective miRNAs and those downregulated in HCC tissues was identified, and the candidate miRNAs were visualized using Venn diagrams. The association between AP003469.2 expression and these miRNAs was investigated further using Spearman correlation. miR-4732-3p displayed the strongest negative correlation and was selected for downstream validation.

Finally, cuproptosis-related genes that were co-expressed with AP003469.2 and negatively correlated with miR-4732-3p were identified as potential downstream targets.

### Cell culture and transfection

The normal human hepatocyte cell line THLE-2 and the HCC cell lines HepG2, Hep3B, Huh-7, and MHCC97-H were obtained from the Chinese Academy of Sciences (Shanghai, China). Cells were cultured in DMEM medium supplemented with 10% fetal bovine serum, 100 units/mL penicillin, and 100 μg/mL streptomycin at 37 °C in a humidified environment containing 5% carbon dioxide. Lentiviral vectors carrying short hairpin RNAs (shRNAs) targeting AP003469.2 or LIPT1, overexpression constructs (OE-AP003469.2, OE-LIPT1), and their negative controls were purchased from LINGSI BIOTECHNOLOGY COMPANY (Wuhan, China). Following the manufacturer’s instructions, miR-4732-3p mimics, inhibitors, and their controls were transfected using Lipofectamine 2000 (Invitrogen, USA).

### Quantitative real-time PCR

Total RNA was isolated from cultured cells and fresh frozen tissues using TRIzol reagent (Invitrogen, USA) following the manufacturer’s instructions. For formalin-fixed, paraffin-embedded (FFPE) samples, RNA was extracted from three sequential 5 μm sections of hepatocellular carcinoma (HCC) tissue employing the RNeasy FFPE Kit (Qiagen, Germany). Complementary DNA was synthesized using the PrimeScript RT Reagent Kit (Takara, China) under the following conditions: reverse transcription was carried out at 37 °C for 15 min, then terminated by incubation at 85 °C for 5 min, after which the samples were maintained at 4 °C. The PowerUp SYBR Green Master Mix (Thermo Fisher Scientific, USA) was used in triplicate for quantitative PCR using an Applied Biosystems 7500 Fast real-time system. snRNA U6 and GAPDH were used as internal references. After normalizing to GAPDH, quantitative analysis was performed using the 2^−ΔΔCt method.

### Colony formation assay

Cells were seeded into 6-well plates at a density of 1×10^6^ cells per well prior to transfection and treatment with drugs. After 24 hours, cells were re−plated in triplicate into 24−well dishes at 2×10^3 cells per well and maintained for 7–14 days, with medium refreshed every three days. When colonies reached more than 50 cells, the cultures were photographed after being fixed for 15 minutes with 4% paraformaldehyde and stained for 20 minutes with 1% crystal violet. To get the colony formation rate, the formula was (number of colonies ÷ number of seeded cells) × 100.

### Transwell migration and invasion assays

Cells were inoculated into the top compartments of inserts that had pore sizes of 8 micrometers in diameter (Corning, USA). For invasion, membranes were precoated with Matrigel before adding cells to the upper compartment (BD Biosciences, USA). The bottom wells were filled with RPMI 1640 that had been supplemented with 10% fetal bovine serum (FBS) in order to establish a chemoattractant gradient. Cells that made it to the membrane’s underside after 24 to 48 hours of incubation were fixed, stained with 0.1% crystal violet for 10 minutes, and then counted under a microscope (Olympus, Japan).

### CCK-8 cell viability assay

The CCK-8 test was performed on HEP3B and HEPG2 cells to assess how Elesclomol and Cu(II)-Elesclomol affect HCC cell viability and to find the best drug concentration for future experiments. Cells undergoing logarithmic growth were placed in 96-well plates at a density of 4,000 cells per well and given 24 hours to adhere. Treatments included graded doses of Elesclomol (1, 2, 5, 10, 20 μM) or Cu(II)-Elesclomol prepared by pre-incubating Elesclomol with CuCl_2_ at a 1:1 molar ratio for 30 minutes, with untreated wells as controls. After a 24-hour exposure period, 10 microliters of CCK-8 solution was added to each well, and the culture plate was then incubated for two hours. The absorbance was measured at 450 nanometers using a microplate reader with blank wells for background subtraction. [(OD_experimental - OD_blank)/(OD_control - OD_blank)] × 100% is the formula used to determine cell viability.

### Copper ion concentration assay

To measure intracellular copper levels, a copper ion detection kit (Elabscience, China) was used on different groups of HCC cells 24 hours after treatment with the optimal concentration (10 μM) of Elesclomol or Cu(II)-Elesclomol (MedChem, USA). The procedure included collecting cell samples, lysing them, and then centrifuging the lysates at 12,000×g to extract the supernatant. Following the manufacturer’s instructions, commercial Copper Assay Kits were used to quantify the supernatant’s copper concentration.

### Subcellular fractionation

The Ensembl gene ID for AP003469.2 was obtained using the UCSC Genome Browser Home (https://genome.ucsc.edu/), and the complete nucleotide sequence was retrieved from NONCODE v7.0 (http://www.noncode.org/). The sequence was input into the lncLocator online prediction tool (http://www.csbio.sjtu.edu.cn/bioinf/lncLocator/) to predict its subcellular localization. High-glucose DMEM with 10% FBS was used to sustain HEPG2 cells throughout the logarithmic growth phase. Subcellular fractionation was performed using the PARIS™ Kit (Ambion, USA) to isolate cytoplasmic and nuclear RNA, and reverse transcription was carried out with the PrimeScript RT reagent Kit (TaKaRa, Japan). AP003469.2 expression was evaluated by quantitative PCR using the SYBR Green qPCR Master Mix (Vazyme, China). Fractionation purity was assessed by Western blot. RIPA buffer mixed with PMSF was used to extract nuclear and cytoplasmic proteins, which were then separated by SDS-PAGE, measured using the BCA test, and probed with anti-tubulin and anti-UAP56 antibodies. Detection employed HRP-conjugated secondary antibodies followed by ECL chemiluminescence. Subcellular localization was corroborated by RNA FISH using a Cy3-labeled probe targeting AP003469.2 (RiboBio, China), together with DAPI counterstaining, and visualization by confocal microscopy on a Zeiss LSM platform.

### Dual-luciferase reporter assay

After being resuscitated, 293T cells (ATCC, USA) were kept in DMEM (Gibco, USA) with FBS (Gibco, China) added. Wild type and mutant fragments of the AP003469.2 3′ UTR and the LIPT1 3′ UTR (Sangon Biotech) were subcloned into the pmiR NC luc2 Rluc dual luciferase reporter vector (Promega, USA). Plasmid combinations containing miR-4732-3p mimics and the corresponding 3′ UTR reporters were delivered into 293T cells using Nanofusion transfection reagent (RiboBio, China). In order to identify if miR-4732-3p bound to AP003469.2 or LIPT1, cells were lysed 24 to 48 hours later using passive lysis solution (Promega, China). Subsequently, firefly/Renilla signal was recorded using luciferase detection reagent II and Stop & Glo^®^ (Promega, China).

### Western blot analysis

Total proteins for Western blot analysis were obtained from cell pellets or tissue samples using RIPA lysis buffer (Biyuntian, China) that included protease inhibitors. The protein concentration was quantified, and samples were denatured before being separated by size via SDS-PAGE. After the proteins had been isolated, they were then placed onto a membrane made of polyvinylidene difluoride (PVDF). The membrane was treated with 5% non-fat milk at room temperature for one hour after the transfer to avoid non-specific binding. The primary antibodies against FDX1 (Baoxin, China), SLC31A1, and HSP70 (Proteintech, China) were diluted 1:1000, and these primary antibodies were applied to the membranes overnight at 4 °C. GAPDH (Abcam, ab9485; 1:5000) was used as the loading control. Following the washing process, a species-matched secondary antibody was added at a 1:2000 dilution, and the mixture was allowed to incubate for an hour at room temperature. Signals were generated by means of increased chemiluminescence, and the intensities of the bands were assessed with the help of ImageJ.

### *In vivo* xenograft experiments

We purchased male BALB/c mice weighing 16–20 g and aged 4–6 weeks from Hunan Slake Jingda Experimental Animal Co., Ltd. (Hunan, China). The animals were divided into two groups at random (n = 3 each group) and given subcutaneous injections of 5×10^6^ HEPG2 cells stably expressing either sh-AP003469.2 or sh-NC. Tumor dimensions were documented every four days using the formula: volume = 0.5 × length × width². The mice were sacrificed after 28 days, and the tumors were taken out, weighed, and ready for copper measurement and immunohistochemical analysis (Ki 67, HSP70, FDX1, SLC31A1). All procedures received approval from the Zunyi Medical University Ethics Committee (Approval No. ZYFY-2024-1-0053).

### Immunohistochemistry

For immunohistochemical examination, xenograft tumors were sectioned, paraffin-embedded, and fixed. The procedure followed established protocols, employing primary antibodies against Ki67 (Abcam, cat. ab15580) at 1:100 dilution, FDX1 (Baoxin, China) at 1:200 dilution, SLC31A1 (Baoxin, China) at 1:200 dilution, and HSP70 (Sanying, China) at 1:400 dilution. In summary, the paraffin sections from xenografts were deparaffinized through incubation at 60 °C for 30 minutes. Rehydration occurred via graded ethanol washes, followed by blocking endogenous peroxidase with 1% hydrogen peroxide in water for 20 minutes. Heat-induced epitope retrieval was performed in citrate buffer (Vector Laboratories, H3300) at boiling temperature for 20 minutes to reveal antigens after sections were cleaned three times with TT solution (500 mM NaCl, 10 mM Tris base, 0.05% Tween 20). Upon reaching ambient temperature, blocking was performed using 2% goat serum combined with 4% BSA in TT buffer for 1 hour. The primary antibodies were left to incubate at a temperature of 4 °C for the duration of the night. The sections were incubated with a biotinylated secondary antibody for a duration of one hour after three rinses in TT buffer, followed by a 1-hour incubation with the Vectastain Elite ABC reagents (Vector Laboratories, PK-6100). Once the additional TT washes were completed, the development process used the DAB substrate system (Vector Laboratories, SK-4100) and involved counterstaining with hematoxylin. Finally, sections underwent dehydration through ethanol and xylene gradients before mounting in a 75% Permount solution (Fisher Scientific, SP15-500) diluted in xylene.

### Statistical analysis

Statistical analysis and data visualization were conducted using R Statistical Software and GraphPad Prism 9. Each experiment was conducted three times, and results are shown as mean ± SD. Either Student’s t-test or one-way ANOVA was used to evaluate whether the results were statistically significant between groups. The examination of the correlation was carried out using Pearson’s coefficient, and a p-value of less than 0.05 was deemed to be statistically significant.

## Results

### Identification and clinicopathological characteristics of the lncRNA AP003469.2

Gene expression analysis of TCGA-LIHC data identified 10 differentially expressed cuproptosis-related genes (|logFC| > 0, FDR < 0.05). Five of these genes (ATP7A, DLAT, LIPT1, PDHA1, and PDHB) were dysregulated and associated with prognosis. To explore the molecular regulatory mechanisms of these five prognostic cuproptosis-related genes and identify functionally relevant lncRNAs, we performed a co-expression network analysis. This analysis identified 175 lncRNAs correlated with the five genes above. We further intersected these 175 co-expressed lncRNAs with 1,582 differentially expressed lncRNAs (**Figure 1A**), yielding 13 candidate lncRNAs (**Figure 1B**). We evaluated the prognostic value of these 13 lncRNAs using Kaplan-Meier survival analysis combined with the log-rank test (*p* < 0.05) and found 8 lncRNAs associated with prognosis (**Figure 1C**). Among these 8 lncRNAs, AP003469.2 showed the highest upregulation (vs. normal liver tissue samples) and most significant expression difference (Additional file 1). Thus, AP003469.2 was selected for further studies.

**Figure 1:**
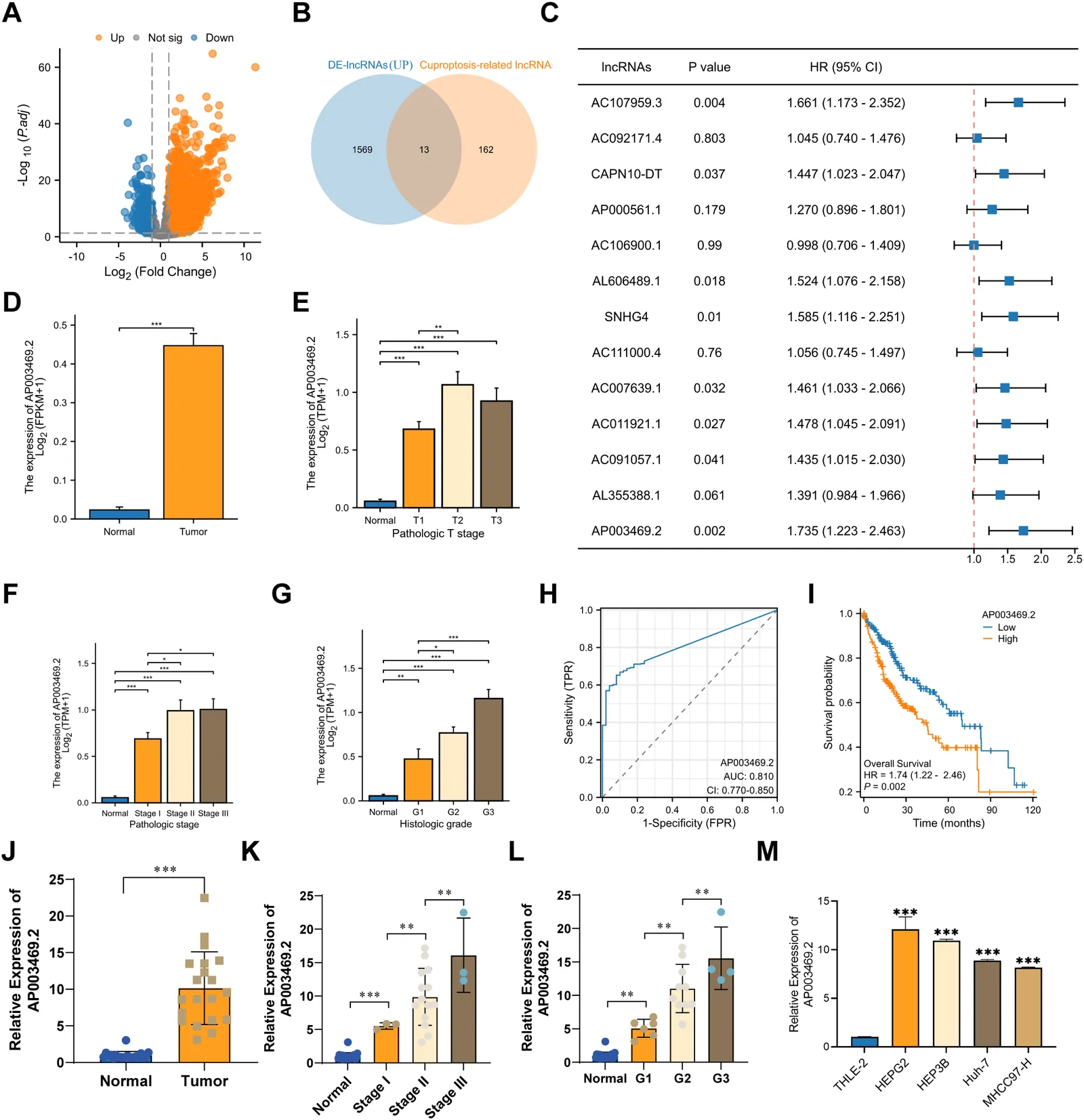
Identification and validation of AP003469.2 as a prognostic biomarker in HCC. **(A)** Volcano graphic illustrating differentially expressed lncRNAs in HCC derived from TCGA data. **(B)** Venn diagram showing the overlap between 1,582 upregulated DE-lncRNAs and 175 cuproptosis-related lncRNAs, yielding 13 candidates. **(C)** Forest plot of univariate Cox analyses for the 13 candidates, displaying associations with overall survival. **(D–G)** AP003469.2 expression patterns in TCGA: higher in tumors than normals **(D)** and positively correlated with pathologic T stage **(E)**, pathologic stage **(F)**, and histologic grade **(G)**. **(H)** ROC curve evaluating the diagnostic performance of AP003469.2 for distinguishing HCC from normal tissue. **(I)** Kaplan–Meier analysis indicating that elevated AP003469.2 expression correlates with worse overall survival in HCC. **(J–L)** qRT-PCR validation in clinical specimens confirming upregulation in tumors **(J)** and positive correlations with advanced pathologic stage **(K)** and histologic grade **(L)**. **(M)** qRT-PCR assessment of AP003469.2 expression across HCC cell lines. (*p < 0.05; **p < 0.01; ***p < 0.001).

AP003469.2 expression was much greater in HCC tissues compared to nearby normal tissues, according to an analysis of TCGA data (**Figure 1D**). Further subgroup analysis confirmed that AP003469.2 expression increased progressively with the advancement of tumor T stage (**Figure 1E**), pathological stage (**Figure 1F**), and histologic grade (**Figure 1G**). ROC showed that AP003469.2 exhibited strong discriminative ability between tumor and normal tissues, with an area under the curve (AUC) of 0.810 (**Figure 1H**). Additionally, Kaplan-Meier survival analysis showed that, in comparison to patients with low expression, those with high AP003469.2 expression had a considerably worse overall survival rate (HR = 1.74, 95% CI: 1.22–2.46) (**Figure 1I**). In line with the TCGA data, the expression of AP003469.2 was higher in clinical HCC tissues than in adjacent normal tissue (**Figure 1J**). Additionally, AP003469.2 expression was positively correlated with both pathological stage and histological grade (**Figures 1K, L**), suggesting a close association with HCC progression. In line with these findings, AP003469.2 was also highly expressed in multiple HCC cell lines (**Figure 1M**). AP003469.2 is further supported by these findings as a possible biomarker for the diagnosis and development of HCC.

To evaluate the clinical relevance of AP003469.2, we divided HCC patients from TCGA data into two groups by median AP003469.2 expression and assessed the association between clinicopathological features and AP003469.2 expression levels. Correlation analysis showed AP003469.2 expression was not associated with age, BMI, or lymph node status. However, elevated AP003469.2 expression correlated significantly with more advanced pathological T stage (*p* = 0.002), higher overall pathological stage (*p* = 0.003), poorer histologic grade (*p* < 0.001), and vascular invasion (*p* = 0.027) (**Table 1**). These results indicate AP003469.2 expression is positively correlated with tumor progression and aggressiveness in HCC.

**Table 1:** Association of AP003469.2 expression with clinicopathological characteristics in 374 HCC patients from TCGA.

Characteristics	Low expression of AP003469.2	High expression of AP003469.2	P value
N	187	187	
Age, n (%)			0.957
<= 60	89 (23.9%)	88 (23.6%)	
> 60	98 (26.3%)	98 (26.3%)	
Pathologic T stage, n (%)			0.002
T1	108 (30.2%)	75 (20.9%)	
T2	38 (10.6%)	57 (15.9%)	
T3	33 (9.2%)	47 (13.1%)	
Pathologic N stage, n (%)			1.000
N0	134 (51.9%)	120 (46.5%)	
N1	2 (0.8%)	2 (0.8%)	
Pathologic stage, n (%)			0.003
Stage I	103 (29.9%)	70 (20.3%)	
Stage II	37 (10.7%)	50 (14.5%)	
Stage III	34 (9.9%)	51 (14.8%)	
BMI, n (%)			0.796
<= 25	91 (27%)	86 (25.5%)	
> 25	80 (23.7%)	80 (23.7%)	
Histologic grade, n (%)			< 0.001
G1	40 (11.2%)	15 (4.2%)	
G2	86 (24.1%)	92 (25.8%)	
G3	51 (14.3%)	73 (20.4%)	
Vascular invasion, n (%)			0.027
No	116 (36.5%)	92 (28.9%)	
Yes	47 (14.8%)	63 (19.8%)	

To confirm these findings, we performed a multivariate analysis using Cox regression, adjusting for confounding factors (patient gender, age, pathological stage, and histological grade). Results showed AP003469.2 remained an independent prognostic factor for HCC (HR = 1.735, 95% CI: 1.223–2.463; Additional file 2), consistent with univariate Cox regression results. In summary, AP003469.2 contributes to HCC progression and serves as an independent predictor of poor prognosis.

### AP003469.2 promotes malignant phenotypes and suppresses cuproptosis

To examine the biological role of lncRNA AP003469.2 in HCC, we modulated its expression in HEPG2 and HEP3B cells and evaluated effects on malignant phenotypes. Functional assays showed AP003469.2 promotes HCC progression. Knockdown of AP003469.2 (sh-AP003469.2) significantly suppressed colony formation vs. the negative control (sh-NC) group, indicating reduced proliferative capacity (**Figure 2A**). Conversely, overexpression of AP003469.2 (OE-AP003469.2) markedly enhanced colony-forming ability vs. the empty vector control (OE-CTRL) (**Figure 2B**). This proliferative effect extended to cell motility. Transwell assays revealed AP003469.2 knockdown significantly impaired migration and invasion of HCC cells, whereas its overexpression substantially enhanced these aggressive behaviors (**Figures 2C, D**).

**Figure 2:**
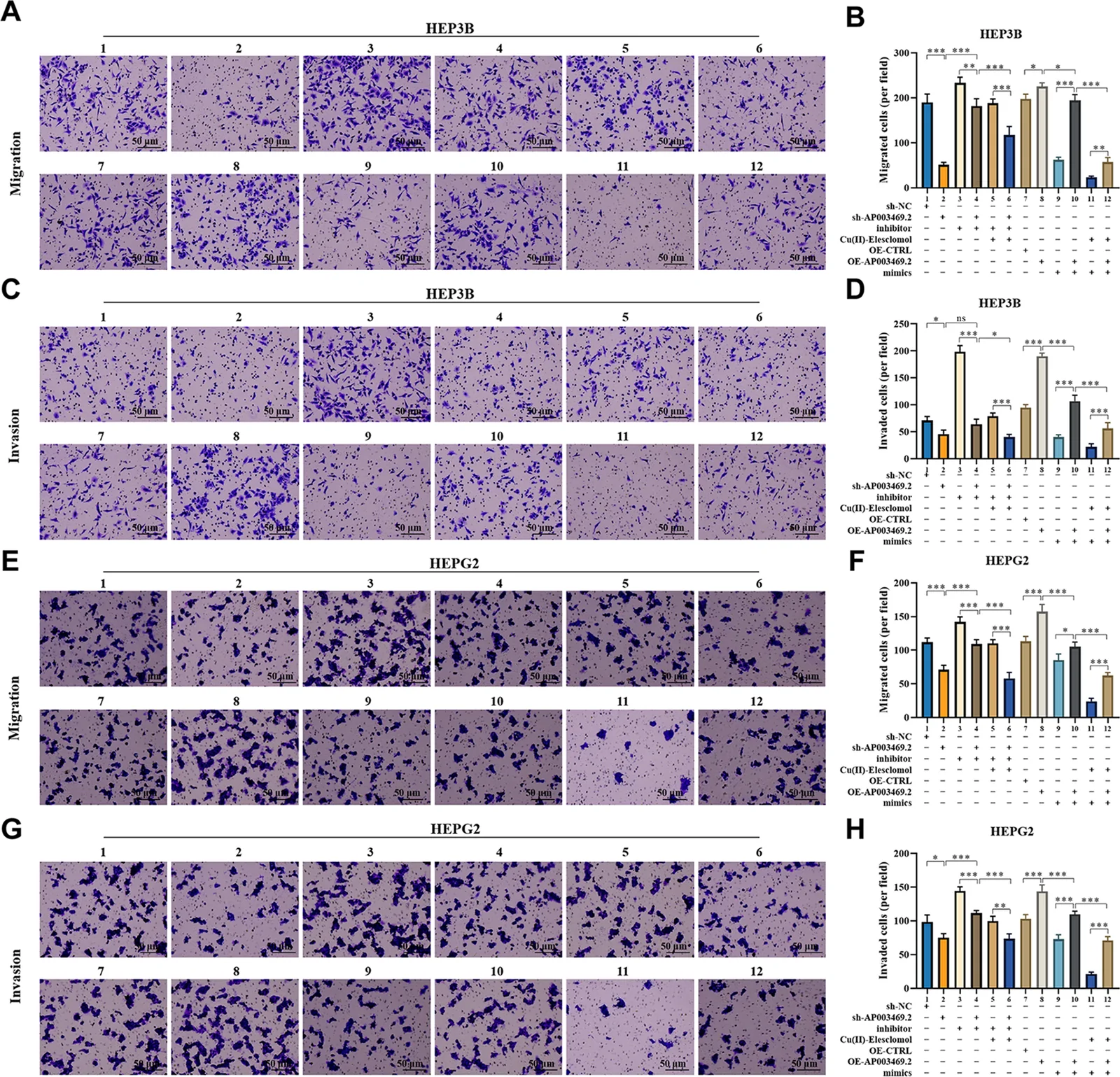
AP003469.2 promotes HCC progression and inhibits cuproptosis. **(A)** Colony formation images and quantification showing that AP003469.2 knockdown reduces proliferative capacity in HEPG2 and HEP3B relative to negative control. **(B)** Colony formation images and quantification indicating that AP003469.2 overexpression enhances proliferation in HEPG2 and HEP3B compared with overexpression control. **(C)** Transwell migration images demonstrating altered migratory ability upon AP003469.2 knockdown or overexpression in HEPG2 and HEP3B (scale bar: 50 µm). **(D)** Transwell invasion images demonstrating altered invasive ability upon AP003469.2 knockdown or overexpression in HEPG2 and HEP3B (Scale bar: 50 µm) **(E)** Cell viability of HEP3B and HEPG2 cells after AP003469.2 knockdown, treated with zVAD-fmk (apoptosis inhibitor), Fer-1 (ferroptosis inhibitor), Cu(II)-Elesclomol (cuproptosis inducer), or TTM (copper chelator). **(F)** Intracellular copper (Cu^2+^) levels in HEP3B and HEPG2 cells. **(G)** Cell viability of HEP3B and HEPG2 cells. **(H–J)** Western blot analysis and corresponding quantification of FDX1 **(H)**, SLC31A1 **(I)**, and HSP70 **(J)** protein levels in HEP3B and HEPG2 cells under different AP003469.2 expression levels and Cu(II)-Elesclomol treatment. (*p < 0.05; **p < 0.01; ***p < 0.001).

To elucidate the mechanisms, we hypothesized that regulated cell death pathways were involved. A cell viability assay showed that AP003469.2 knockdown-induced reduction in cell viability was partially but significantly rescued by the cuproptosis inhibitor tetrathiomolybdate (TTM); the ferroptosis inhibitor Ferrostatin-1 (Fer-1) had no such restorative effect (**Figure 2E**). This result strongly implicated cuproptosis as the key downstream pathway. We then directly measured intracellular free copper ion (Cu^2+^) levels. AP003469.2 knockdown significantly increased intracellular Cu^2+^ accumulation, and this effect was further exacerbated by treatment with the cuproptosis inducers Elesclomol or Cu(II)-Elesclomol (10 μM) (**Figure 2F**). In contrast, AP003469.2 overexpression lowered basal Cu^2+^ levels and blocked Cu^2+^ accumulation induced by these agents. These changes in copper homeostasis directly affected chemosensitivity. **Figure 2G** illustrates that the knockdown of AP003469.2 enhanced the sensitivity of HCC cells to Elesclomol and Cu(II)-Elesclomol, resulting in a significant reduction in cell viability. Conversely, AP003469.2 overexpression conferred significant resistance, partially rescuing cells from the cytotoxicity of these cuproptosis inducers.

We investigated the key proteins’ expression in the cuproptosis pathway by Western blot analysis to understand how AP003469.2 inhibits cuproptosis.

In HEPG2 and HEP3B cells, knockdown of AP003469.2 reduced the expression of cuproptosis regulator FDX1 while increasing copper transporter SLC31A1 and stress marker HSP70 levels. Cu(II)-Elesclomol treatment enhanced these effects. Conversely, AP003469.2 overexpression upregulated FDX1 but downregulated SLC31A1 and HSP70, and Cu(II)-Elesclomol reversed these changes (**Figures 2H–J**). These findings indicate that AP003469.2 modulates cuproptosis in HCC cells by regulating FDX1, SLC31A1, and HSP70 expression.

Based on the aforementioned findings, lncRNA AP003469.2 may promote the malignancy of HCC by suppressing cuproptosis through the coordinated regulation of intracellular copper levels and the expression of key cuproptosis-associated proteins.

### AP003469.2 modulates cuproptosis and HCC cell proliferation, migration, and invasion

To investigate AP003469.2’s role in regulating cuproptosis and HCC progression, we manipulated its expression in HEP3B and HEPG2 cells and treated the cells with Cu(II)-Elesclomol (10 μM). Under Cu(II)-Elesclomol treatment, AP003469.2 knockdown significantly increased intracellular Cu^2+^ levels in both cell lines (**Figures 3A, B**); in contrast, its overexpression markedly reduced Cu^2+^ accumulation vs. control cells (p < 0.05). Colony formation assays (**Figures 3C–E**) showed AP003469.2 knockdown suppressed HCC cell proliferation under copper-induced stress, while its overexpression promoted colony formation (p < 0.05); representative images confirmed these quantitative results. Transwell assays (**Figures 3F–K**) revealed AP003469.2 silencing significantly decreased HCC cell migration and invasion, whereas overexpression enhanced these capabilities. Cu(II)-Elesclomol treatment further modulated these effects, indicating AP003469.2 regulates HCC cell metastatic potential via copper-dependent mechanisms.

**Figure 3:**
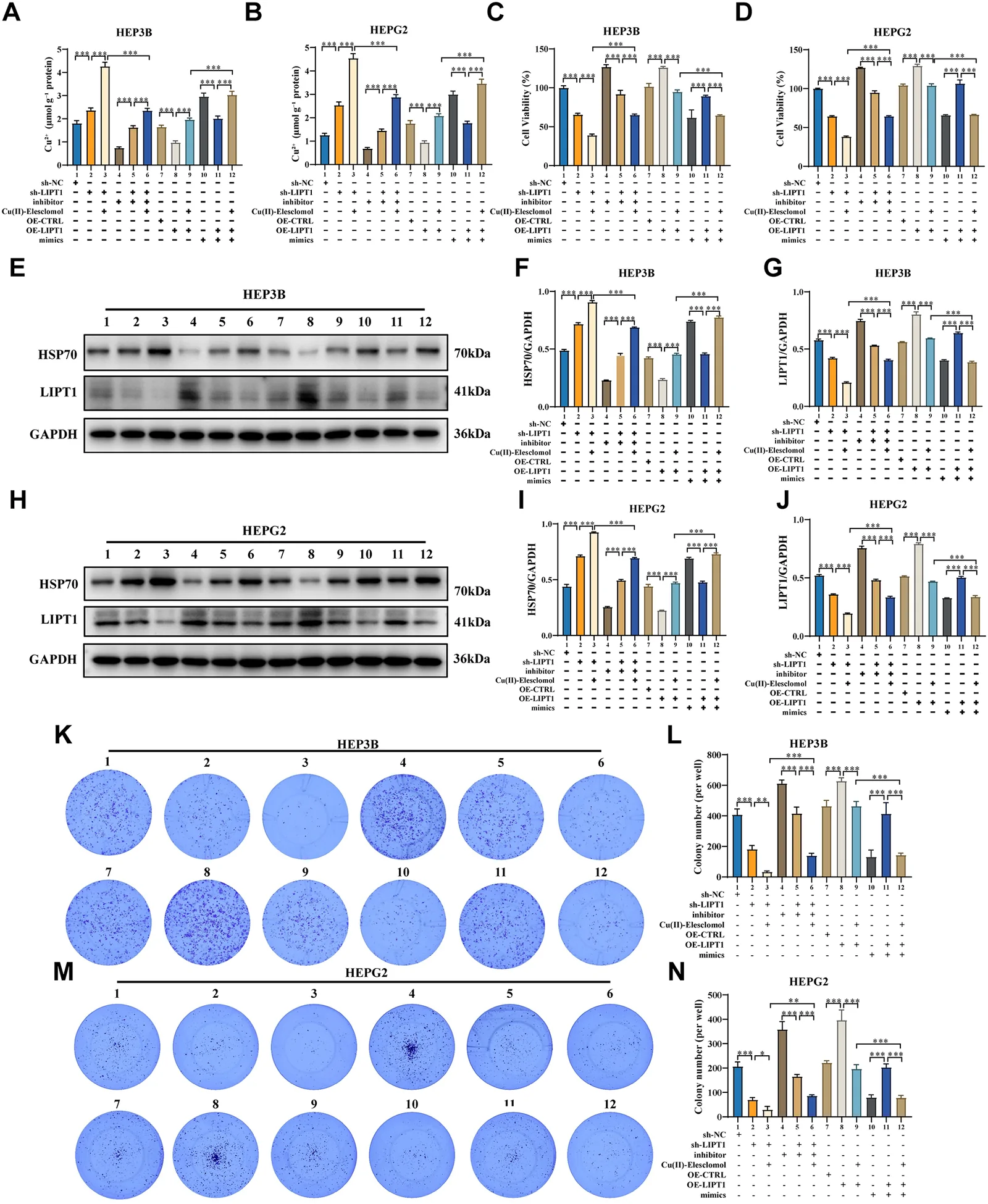
AP003469.2 regulates intracellular copper to increase HCC cell proliferation, migration, and invasion. **(A)** Intracellular copper (Cu^2+^) levels in HEP3B under different experimental conditions. **(B)** Intracellular copper (Cu^2+^) levels in HEPG2 cells under different experimental conditions. **(C, D)** Quantification of colony numbers in colony formation assays for HEP3B **(C)** and HEPG2 **(D)** cells, demonstrating the effects of AP003469.2 knockdown or overexpression and Cu(II)-Elesclomol treatment on cell proliferation. **(E)** Representative pictures of the HEP3B and HEPG2 cell colony formation tests that match the information in **(C, D)**. **(F, H)** Quantification of migrated cells per field in transwell migration assays for HEP3B **(F)** and HEPG2 **(H)** cells, showing the impact of AP003469.2 knockdown/overexpression and Cu(II)-Elesclomol treatment on cell migratory ability. **(G, I)** Quantification of invaded cells per field in transwell invasion assays for HEP3B **(G)** and HEPG2 **(I)** cells, illustrating the effects of AP003469.2 knockdown/overexpression and Cu(II)-Elesclomol treatment on cell invasive capacity. **(J, K)** Migration and invasion images for HEP3B and HEPG2 cells under different conditions. (scale bar: 50 µm) (*p < 0.05; **p < 0.01; ***p < 0.001). (Scale bar: 50 µm).

In the xenograft mouse model (**Figure 4A**), inhibiting AP003469.2 significantly suppressed *in vivo* tumor growth. This was evidenced by reduced tumor size, weight (**Figures 4B–D**), and volume (**Figure 4E**), with no significant difference in mouse body weight (**Figure 1F**). Mechanistically, AP003469.2 inhibition altered copper homeostasis in tumors, increasing total copper concentration (**Figure 4G**). Western blot (**Figure 5H**) and immunohistochemical analyses (**Figures 4I, J**) further showed that AP003469.2 inhibition upregulated HSP70 and SLC31A1 expression while downregulating FDX1. Immunohistochemistry also confirmed a significant reduction in cell proliferation (Ki67 expression) in AP003469.2-inhibited tumors (**Figures 4I, J**). These *in vivo* results align with earlier *in vitro* findings.

**Figure 4:**
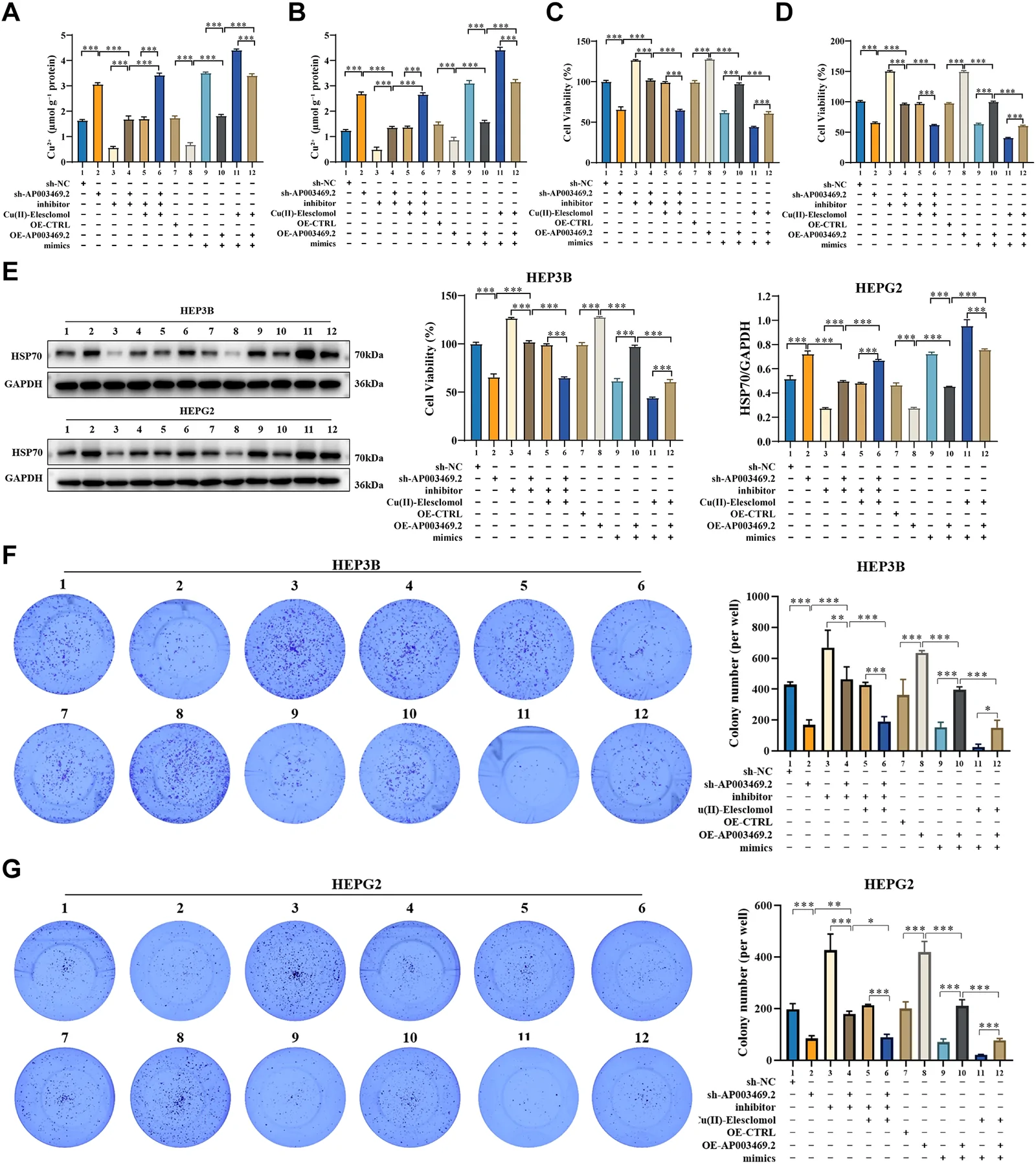
AP003469.2 knockdown inhibits HCC xenograft tumor growth and alters cuproptosis-related protein expression *in vivo*. **(A)** Diagram depicting bilateral subcutaneous injections of sh-NC and sh-AP003469.2 modified cells into nude mice to generate control and knockdown tumors, respectively. **(B)** Gross appearance of excised tumors from the sh-NC and sh-AP003469.2 groups, demonstrating the difference in tumor size. **(C)** Quantification of tumor weights (mg) from the sh-NC and sh-AP003469.2 groups. **(D)** Growth curves illustrating tumor volume (mm³) measured over 28 days in the sh-NC and sh-AP003469.2 groups. **(E)** Body weight (g) of nude mice measured over 28 days after cell injection. **(F)** Relative expression of copper concentration in tumor tissues derived from sh-NC and sh-AP003469.2 groups. **(G)** Western blot analysis and corresponding quantification of HSP70, FDX1, and SLC31A1 protein levels in tumor tissues from sh-NC and sh-AP003469.2 groups. GAPDH served as an internal loading control. **(H)** Quantification of the positive staining area (%) for Ki67, HSP70, FDX1, and SLC31A1 in tumor sections from sh-NC and sh-AP003469.2 groups, as determined by immunohistochemistry. **(I)** Representative H&E images (left) and immunohistochemical staining for Ki-67, HSP70, FDX1, and SLC31A1 in tumors from sh-NC versus sh-AP003469.2 groups (magnification ×200; scale bar 100 µm). (*p < 0.05; **p < 0.01; ***p < 0.001; ****p < 0.0001). sh-NC, negative control. sh-AP003469.2, AP003469.2 knockdown.

**Figure 5:**
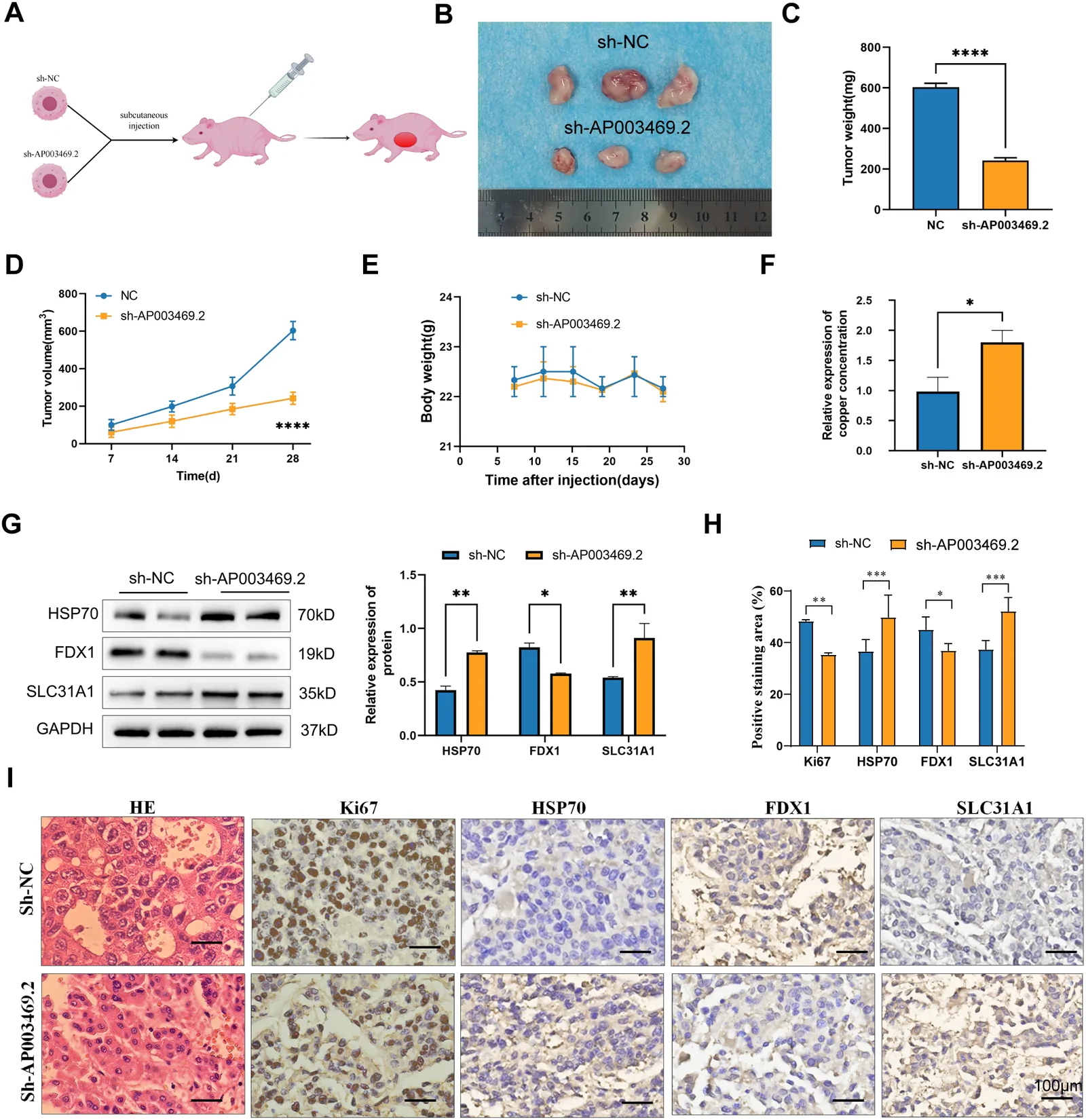
AP003469.2 modulates cuproptosis and cell proliferation via miR-4732-3p. **(A)** Intracellular Cu^2+^ concentration in HEP3B under various treatment conditions. **(B)** Intracellular Cu^2+^ concentration in HEPG2 under various treatment conditions. **(C, D)** Cell viability (percentage) in HEP3B **(C)** and HEPG2 **(D)** cells after the indicated treatments. **(E)** Western blot showing HSP70 protein levels and corresponding densitometric quantification in HEP3B and HEPG2 cells under the specified treatment conditions. **(F)** Colony formation assays in HEP3B under various treatment conditions. **(G)** Colony formation assays in HEPG2 under various treatment conditions. (*p < 0.05, **p < 0.01, ***p < 0.001).

The data above indicate that AP003469.2 is essential for controlling cuproptosis and promoting HCC cell invasion, migration, and proliferation.

### Subcellular localization of AP003469.2 and its role as a molecular sponge through direct binding to miR-4732-3p

According to lncLocator (http://www.csbio.sjtu.edu.cn/bioinf/lncLocator/), AP003469.2 (ENSG00000253395.1, 430 nt) is primarily located in the cytoplasm (score 0.4715) but also shows some nuclear presence (score 0.4086) (**Supplementary Figure 1A**). FISH further revealed predominant cytoplasmic localization of AP003469.2, largely separate from DAPI-stained nuclei (**Supplementary Figures 1B, C**). These results indicate AP003469.2 primarily resides in the cytoplasm, suggesting potential roles in cytoplasmic signaling or miRNA sponging.

lncRNAs act as molecular sponges to regulate microRNAs, which in turn affect downstream target genes (37, 38). To predict potential miRNAs targeted by lncRNA AP003469.2, we used the ceRNA regulatory mechanism. miRNA data were filtered via the “limma” package, yielding 364 differentially expressed miRNAs (268 upregulated, 96 downregulated; **Supplementary Figure 2A**). Multivariate Cox regression analysis identified 219 survival-associated miRNAs, 43 of which were statistically significant (HR < 1). A Venn diagram of the intersection between these 43 prognostic miRNAs and 96 downregulated miRNAs revealed 3 candidates (**Supplementary Figure 2B**). MiR-4732-3p and AP003469.2 had the highest negative correlation, according to correlation analysis between these miRNAs and AP003469.2 (R = -0.376; **Supplementary Figures 2C–E**). Therefore, miR-4732-3p was chosen for further investigation.

Next, we analyzed the clinical implications of miR-4732-3p expression in HCC. TCGA data further validated decreased miR-4732-3p expression in tumors, with progressive decline across advanced pathological stages and higher histological grades (**Supplementary Figures 2F–H**). ROC curve analysis showed miR-4732-3p had moderate diagnostic value for HCC (**Supplementary Figure 2I**), and an inverse correlation with AP003469.2 was observed (**Supplementary Figure 2J**). According to qRT–PCR validation, miR-4732-3p expression was consistently found to be lower in clinical tumor tissues than in the surrounding nontumor tissues (**Supplementary Figure 2K**). In the xenograft tumors, the knockdown of AP003469.2 also led to an increase in miR-4732-3p expression (**Supplementary Figure 2L**). Functional assays demonstrated that AP003469.2 overexpression significantly suppressed miR-4732-3p levels, while miR-4732-3p mimics or inhibitors effectively modulated its expression in HCC cell lines (**Supplementary Figures 2M, N**).

Finally, to examine the relationship between miR-4732-3p and AP003469.2, we predicted potential binding sites using RNAhybrid. The analysis showed strong binding affinity (binding free energy < -24.6 kcal/mol; **Supplementary Figures 2O, P**). Dual-luciferase reporter assays validated this prediction, confirming direct binding of miR-4732-3p to the 3’UTR of AP003469.2 (**Supplementary Figure 2Q**). These results demonstrate the functional interaction between the two RNA molecules.

### AP003469.2 regulates miR-4732-3p to modulate cuproptosis and cell proliferation, migration and invasion in HCC cells

To investigate whether AP003469.2 regulates miR-4732-3p to modulate cuproptosis and proliferation in HCC, we assessed the intracellular copper concentration, cell viability, stress response, and clonogenic capacity under various treatments. In both HEP3B and HEPG2 cells, AP003469.2 knockdown significantly increased intracellular copper accumulation, a result that was largely reversed by miR-4732-3p suppression. Conversely, AP003469.2 overexpression reduced copper levels, whereas miR-4732-3p mimics abrogated this effect. Under Cu(II)-Elesclomol challenge, AP003469.2 suppression synergized with miR-4732-3p activation to further enhance copper overload, whereas AP003469.2 overexpression conferred resistance to copper accumulation induced by miR-4732-3p mimics (**Figures 5A, B**). Consistently, cell viability assays revealed that AP003469.2 knockdown markedly impaired cell growth, whereas treatment with the miR-4732-3p inhibitor partially reversed this effect. AP003469.2 overexpression promoted cell survival, whereas cotransfection with miR-4732-3p mimics abrogated this growth advantage. Importantly, under Cu(II)-Elesclomol treatment, AP003469.2 knockdown sensitized HCC cells to copper-induced death, while its overexpression mitigated the cytotoxicity driven by miR-4732-3p (**Figures 5C, D**). Western blot analysis revealed that AP003469.2 depletion increased HSP70 expression, which is indicative of cellular stress, whereas its overexpression reduced HSP70 levels (**Figure 5E**). Additionally, the knockdown of AP003469.2 dramatically decreased the capacity to form colonies, as demonstrated by clonogenic assays, although this effect was partially mitigated by inhibiting miR-4732-3p. Conversely, AP003469.2 overexpression promoted clonogenic growth, but this effect was suppressed by the miR-4732-3p mimics (**Figures 5F, G**). These findings suggest that AP003469.2 regulates copper homeostasis and cell survival through miR-4732-3p, thereby modulating cellular sensitivity to cuproptosis.

To examine the impact of AP003469.2 on the advancement of HCC, we conducted migration and invasion experiments. AP003469.2 knockdown led to a marked reduction in HCC cell migration and invasion compared to the control group. miR-4732-3p inhibitor co-treatment partially rescued these suppressive effects. AP003469.2 overexpression enhanced both migration and invasion, but miR-4732-3p mimics markedly impaired these functions. Under Cu(II)-Elesclomol stimulation, AP003469.2 knockdown synergized with miR-4732-3p activation to further suppress cell motility. The suppressive effects of miR-4732-3p mimics were weakened by the overexpression of AP003469.2 (**Figure 6**).

**Figure 6:**
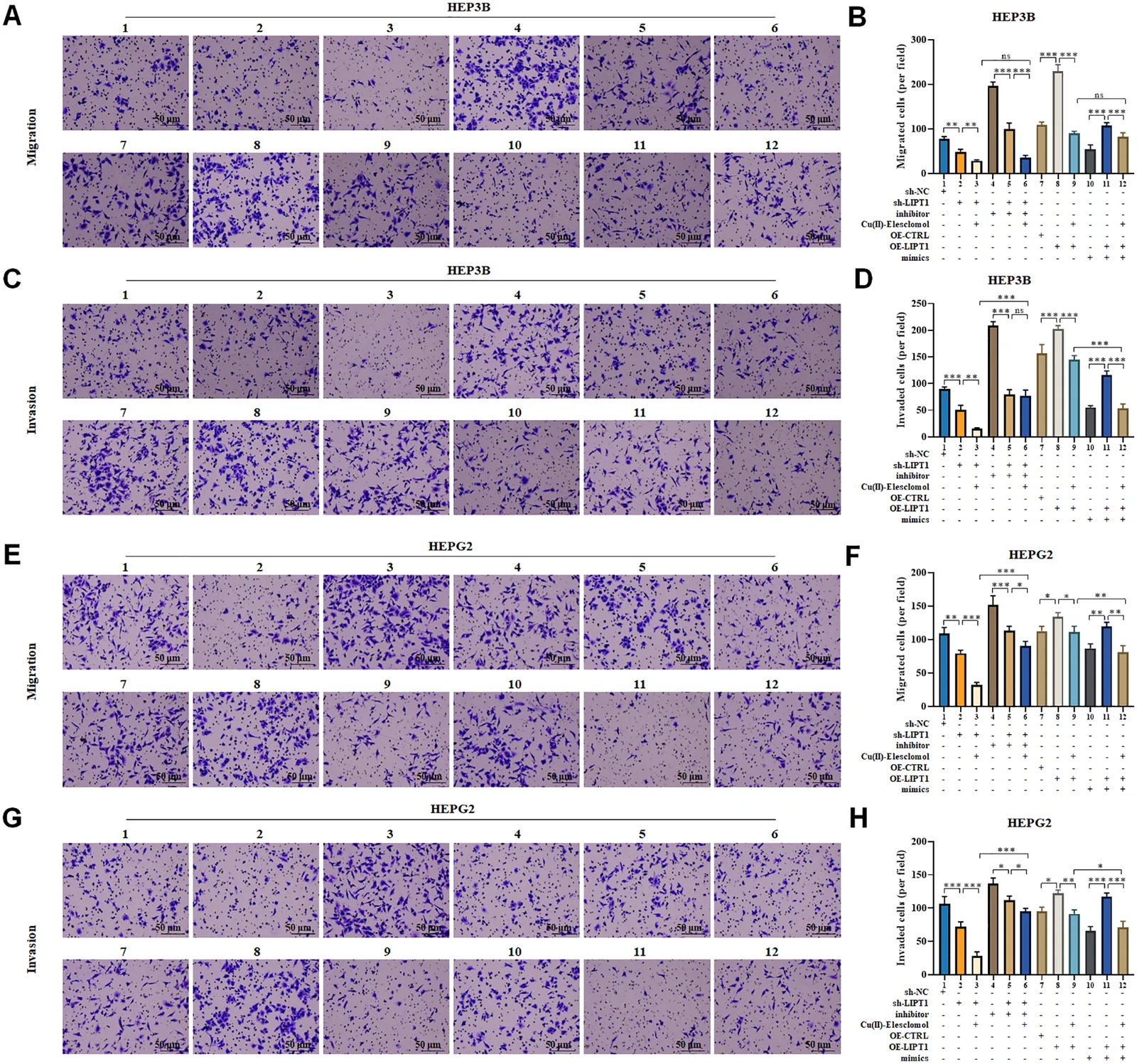
AP003469.2 regulates migration and invasion via miR-4732-3p. **(A, E)** present representative images of migration assays in HEP3B and HEPG2 cells, respectively, depicting cells that have successfully migrated through the membrane. **(B, F)** provide the quantification of migrated HEP3B and HEPG2 cells per field, respectively, expressed as mean ± SD. **(C, G)** display representative images of Transwell invasion assays for HEP3B and HEPG2 cells, showing cells that have invaded through the Matrigel-coated membrane. **(D, H)** quantify the invaded HEP3B and HEPG2 cells per field, respectively, also presented as mean ± SD. A 50 μm scale bar is included in all microscopic images. (*p < 0.05, **p < 0.01, ***p < 0.001).

The findings demonstrate that AP003469.2 controls the susceptibility to cuproptosis and the malignant phenotype of HCC via regulating miR-4732-3p–dependent copper balance, therefore underlining its functional involvement in the evolution of HCC.

### AP003469.2 modulates LIPT1 expression via miR-4732-3p in HCC

A significant correlation between AP003469.2 and LIPT1 was revealed through the co-expression analysis of lncRNAs and cuproptosis-related genes (**Supplementary Figure 3A**). Subsequent analyses revealed that LIPT1 was upregulated in tumor tissues and that its level increased with increasing pathological stage and histological grade in the TCGA data (**Supplementary Figures 3B–D**). ROC analysis showed that LIPT1 had robust diagnostic performance (AUC = 0.853; **Supplementary Figure 3E**). Kaplan-Meier survival analysis indicated that elevated LIPT1 expression was associated with poor overall survival (**Supplementary Figure 3F**). Consistent with the TCGA data, LIPT1 expression was higher in HCC tissues and various HCC cell lines compared to nearby normal tissues and normal liver cell lines (**Supplementary Figures 3G, H**). Similar conclusions were drawn in the xenograft tumor model (**Supplementary Figures 3I, J**). According to correlation analysis, LIPT1 expression had an unfavorable relationship with miR-4732-2p and a positive correlation with AP003469.2 (**Supplementary Figures 3K–N**). Predicted binding between LIPT1 and miR−4732−3p was obtained using RNAhybrid and subsequently validated by dual−luciferase reporter assays, confirming a direct interaction (**Supplementary Figures 3O–Q**). The above results suggest that AP003469.2/miR-4732-3p/LIPT1 may represent a functional ceRNA regulatory network in HCC.

Subsequently, we confirmed that the functional interaction between AP003469.2, miR-4732-3p, and LIPT1 in HCC cells. In HEP3B cells, silencing AP003469.2 significantly reduced LIPT1 mRNA expression, whereas inhibition of miR-4732-3p partially reversed this effect. Conversely, the overexpression of AP003469.2 markedly upregulated LIPT1, and the effect was further strengthened in the presence of the miR-4732-3p mimics (**Supplementary Figure 4A**). Similar expression patterns were consistently observed in HEPG2 cells (**Supplementary Figure 4B**). Western blot analysis demonstrated that the knockdown of AP003469.2 reduced LIPT1 expression, a reduction that was restored by the inhibition of miR-4732-3p. In contrast, AP003469.2 overexpression increased LIPT1 protein expression, which decreased after cotransfection with the miR-4732-3p mimics (**Supplementary Figure 4C**). Quantitative analysis of protein levels confirmed these results in both HEP3B and HEPG2 cells (**Supplementary Figures 4D, E**). These findings demonstrate that AP003469.2 boosts LIPT1 expression by acting as a sponge for miR-4732-3p, establishing the AP003469.2/miR-4732-3p/LIPT1 regulatory axis in HCC.

### miR-4732-3p targets LIPT1 to modulate cuproptosis and malignant phenotype in HCC cells

In HEP3B and HEPG2 cells, knockdown of LIPT1 led to a remarkable increase in intracellular concentration of copper ions, decreased cell viability and induction of the stress marker HSP70, whereas overexpression of LIPT1 had the opposite effects (**Figures 7A–J**). Cu(II)-Elesclomol treatment further amplified copper accumulation and stress responses, even in LIPT1-overexpressing cells. Inhibition of miR-4732-3p increased LIPT1 expression and reversed these effects, whereas miR-4732-3p mimics suppressed LIPT1 and exacerbated copper-induced stress, indicating that miR-4732-3p regulates copper homeostasis primarily through LIPT1.

**Figure 7:**
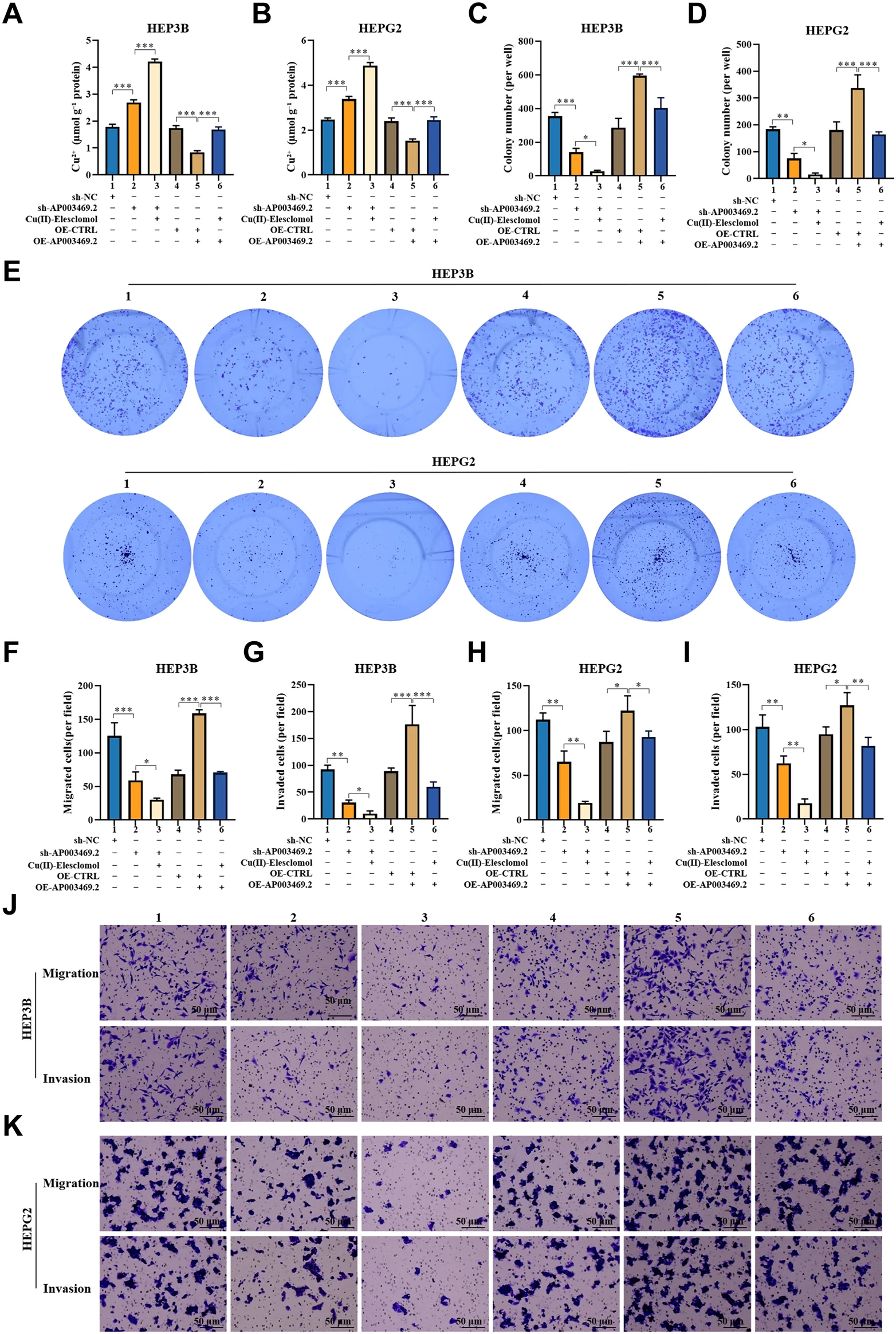
miR-4732-3p targets LIPT1 to modulate cuproptosis and cell proliferation. **(A)** Intracellular Cu^2+^ levels in HEP3B cells subjected to different treatments. **(B)** Intracellular Cu^2+^ levels in HEPG2 cells subjected to different treatments. **(C)** Viability of HEP3B cells measured after treatment by CCK-8. **(D)** Viability of HEPG2 cells assessed following treatment by CCK-8. **(E)** Protein expression of HSP70, LIPT1, and GAPDH in HEP3B cells under various conditions by western blot. **(F)** Densitometric analysis of HSP70 expression normalized to GAPDH in HEP3B cells, derived from the Western blot data in **(E)**. **(G)** Densitometric quantification of LIPT1 expression relative to GAPDH in HEP3B cells, calculated from the Western blot shown in **(E)**. **(H)** Western blot detection of HSP70, LIPT1, and GAPDH protein levels in HEPG2 cells. **(I)** Relative HSP70 expression normalized to GAPDH in HEPG2 cells, quantified from the blot presented in **(H)**. **(J)** Relative LIPT1 expression normalized to GAPDH in HEPG2 cells, quantified from the blot presented in **(H)**. **(K)** Representative images of colony formation assays demonstrating the proliferative ability of HEP3B cells exposed to different treatments. **(L)** Quantitative analysis of colony counts per well for HEP3B cells obtained from the assay results in **(K)**. **(M)** Representative colony formation assay images illustrating the proliferative potential of HEPG2 cells under the indicated treatments. **(N)** Quantitative evaluation of colony numbers per well for HEPG2 cells based on the assay shown in **(M)**. (**p < 0.01, ***p < 0.001.).

Functionally, LIPT1 knockdown markedly impaired clonogenic growth, migration, and invasion, with Cu(II)-Elesclomol further reinforcing these inhibitory effects (**Figures 7K–N**, **8A–H**). Conversely, LIPT1 overexpression enhanced colony formation and metastatic behavior and partially rescued the suppression induced by miR-4732-3p. However, copper overload abrogated the pro-growth and pro-migratory effects of LIPT1.

**Figure 8:**
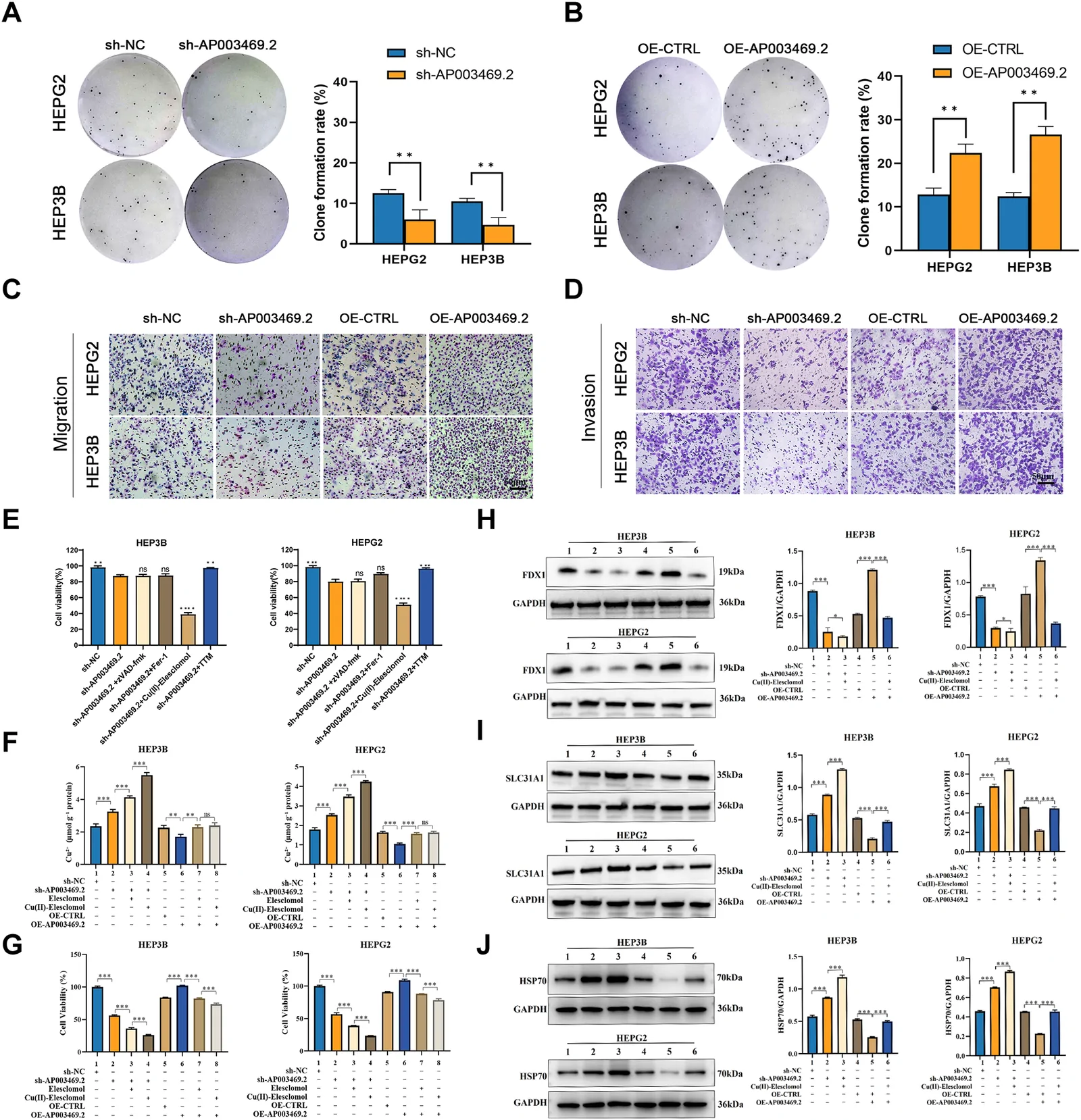
miR-4732-3p targets LIPT1 to modulate cuproptosis and cell migration and invasion. **(A, B)** HEP3B cell migration following different treatments. **(C, D)** HEP3B cell invasion following different treatments. **(E, F)** HEPG2 cell migration following different treatments. **(G, H)** HEPG2 cell invasion following different treatments. Scale bar = 50 µm. (*p < 0.05, **p < 0.01, ***p < 0.001.).

The results indicate that miR-4732-3p suppresses LIPT1 expression,–resulting in elevated intracellular copper levels and triggering stress responses in HCC cells. This stress response consequently impairs cell proliferation and metastatic capacity by interfering with major oncogenic signaling pathways. Furthermore, copper-induced stress acts as a strong enhancer of this regulatory cascade, consequently enhancing miR-4732-3p’s tumor-suppressive effect on the development of HCC.

## Discussion

Our study identified lncRNA AP003469.2 as markedly upregulated in HCC tissues and cell lines via TCGA sequencing data analysis and qRT–PCR validation. Analysis of clinical characteristics and follow-up data showed high AP003469.2 expression correlated significantly with a more advanced TNM stage and a worse prognosis. These findings suggest AP003469.2 may be a novel therapeutic or diagnostic biomarker for HCC. To explore its biological function, we conducted functional assays to assess the impact of AP003469.2 on HCC malignant phenotypes. The findings showed that increased expression of AP003469.2 enhanced the malignant phenotype of HCC cells; its knockdown exerted inhibitory effects. Mechanistically, AP003469.2 enhances HCC progression by sponging miR-4732-3p. Specifically, the sponging of miR-4732-3p by AP003469.2 leads to the upregulation of LIPT1, which in turn maintains copper homeostasis, reduces proteotoxic stress, and suppresses cuproptosis sensitivity. Consistent with the *in vivo* findings from functional assays, additional animal studies further confirmed that AP003469.2 facilitates *in vivo* tumor growth. Collectively, our results identify the AP003469.2/miR-4732-3p/LIPT1 axis as a key driver of HCC progression and highlight AP003469.2 as a promising diagnostic indicator and therapeutic target to sensitize HCC to cuproptosis-inducing agents.

The process of cuproptosis involves regulated cell death that is dependent on copper, driven by the clustering of mitochondrial proteins and the stress from toxic proteins (10, 39). Previous research has revealed that some genes associated with cuproptosis, including FDX1, ATP7A, ATP7B, DLAT, SLC31A1, and LIPT1, display abnormal expression patterns in a wide range of cancers, where they are closely connected to metastatic spread and resistance to chemotherapy (13, 40, 41). For instance, FDX1 has emerged as one of the most significant predictive biomarkers for cuproptosis sensitivity (14). High FDX1 expression sensitizes cells to copper toxicity by facilitating iron-sulfur cluster disruption and enhancing mitochondrial dysfunction. Research demonstrates that FDX1 expression levels can stratify cancer patients into different risk groups, with higher-expressing tumors showing better responses to copper ionophore treatments (36). SLC31A1 serves as the predominant copper uptake mechanism in mammalian cells (42). Multiple studies have demonstrated that SLC31A1 expression levels directly correlate with cellular copper accumulation and cuproptosis sensitivity (43, 44). ATP7A and ATP7B function as copper efflux pumps and represent critical resistance biomarkers (43). Overexpression of these transporters enables cancer cells to maintain copper homeostasis under stress conditions, thereby conferring resistance to cuproptosis induction (45). In HCC specifically, elevated LIPT1 expression enhances mitochondrial resilience, promoting tumor growth and resistance to copper overload, with correlations to advanced stages and poor survival (15). In addition, HSP70 functions as a major protective factor against cuproptosis by preventing protein aggregation and maintaining cellular proteostasis under copper stress (46). Elevated HSP70 expression in tumors often correlates with reduced cuproptosis sensitivity and worse therapeutic outcomes (47). This makes HSP70 an important biomarker for predicting treatment resistance. Our research extends these insights by revealing that AP003469.2 inhibits cuproptosis in HCC cells, reducing copper accumulation and modulating FDX1, SLC31A1, and HSP70 expression, thereby fostering malignant phenotypes. This elucidation of cuproptosis suppression in HCC underscores its mechanistic significance, indicating that reinstating cuproptosis might be a new approach to halt tumor growth and enhance treatment results.

lncRNAs play a significant role as regulators in the progression of HCC, particularly through their intricate interactions with ion concentration, ion channels, and second messengers, which collectively influence cellular processes such as cuproptosis (48–50). For instance, it has been shown that lncRNA RP11-295G20.2 stimulates the development of HCC by causing the tumor suppressor PTEN to become unstable via lysosomal degradation. This indirectly impacts downstream signaling pathways that include AKT and FOXO3a, which are essential for the body’s reactions to ion imbalances (51). Similarly, LINC01234 has been implicated in reprogramming aspartate metabolism by inhibiting the expression of ASS1, a key enzyme in the urea cycle, thereby influencing mTORC1 signaling and cellular energy consumption, which are tightly regulated by ion concentrations (52). The study emphasizes the capacity of lncRNAs to serve as primary controllers of metabolic and signaling pathways vital for the survival and proliferation of cancer cells. In addition, lncRNAs significantly influence the cellular signaling pathways that control cancer development. For example, the lncRNA H19 is linked to the regulation of NFAT signaling, a pathway that is highly sensitive to fluctuations in calcium ions, and it is downregulated in failing hearts but upregulated in certain cancers (53). This suggests that lncRNAs may modulate calcium-dependent signaling cascades, which are important for functions like cell proliferation, migration, and cell death. Additionally, lncRNA TARID has been found to regulate Tcf21, a transcription factor associated with transforming growth factor-β signaling, influenced by ion channel activity and secondary messenger systems (54). These interactions point out the ability of lncRNAs to fine-tune cellular responses to ion concentration changes, thereby influencing cancer progression and therapeutic resistance. In the present study, we found that cytoplasmic AP003469.2 enhances HCC aggressiveness by suppressing cuproptosis via altered copper ion levels and related protein regulation, building on these ion-centric roles to uncover its specific involvement in cell death resistance. This discovery highlights the broader implications of lncRNA-ion interactions in HCC, paving the way for therapies targeting such networks to reinstate cuproptosis and halt disease advancement.

According to the ceRNA hypothesis, lncRNAs function as molecular sponges that sequester miRNAs to influence downstream target gene expression (55, 56). This ceRNA mechanism has been increasingly recognized as a critical regulatory layer in tumorigenesis, metastasis, and drug resistance (57, 58). LINC01133, for example, functions as a sponge for miR-199a-5p, leading to the elevation of Snail expression. This promotes the epithelial-mesenchymal transition (EMT) and speeds up the metastasis of HCC (59). Similarly, MAGI2-AS3 acts as a ceRNA for miR-96 and miR-182, protecting target mRNAs like Decorin (DCN) from miRNA-mediated repression, which plays a vital role in preventing the development and spread of tumors in breast cancer (60). In the study, we used bioinformatics tools, including RNAhybrid, miRDB, and LNCipedia, to predict the AP003469.2/miR-4732-3p axis. The findings revealed that LIPT1, an essential gene involved in cuproptosis, is predicted to be a downstream target of miR-4732-3p. Abnormal expression of LIPT1 plays a key role in tumorigenesis and tumor progression. For example, increased levels of LIPT1 are associated with accelerated tumor growth and an unfavorable prognosis in HCC, where it modulates mitochondrial metabolism and cell - death pathways (15). Furthermore, recent studies indicate that targeting LIPT1 can sensitize cancer cells to cuproptosis inducers, inhibiting proliferation and metastasis in HCC models (61). Our analysis revealed that elevated levels of miR-4732-3p suppressed LIPT1 expression, while reduced miR-4732-3p led to its upregulation. Dual-luciferase reporter assays confirmed that miR-4732-3p directly binds to the 3′-UTR of LIPT1. We also showed that LIPT1 levels were higher in HCC tissues and cell lines, showing a negative relationship with miR-4732-3p and a positive relationship with AP003469.2. Notably, prior work by Qi et al. demonstrated that LINC00638 functions as a ceRNA for miR-4732-3p in HCC, relieving its suppression of ULBP1 and thereby facilitating immune escape via PD-L1 upregulation in tumors with high tumor mutation burden (62). This finding supports the notion that miR-4732-3p can be regulated by lncRNAs in a sponge manner and plays a pivotal role in the tumor immune microenvironment of HCC, which is consistent with the regulatory mechanism we identified involving lncRNA AP003469.2 and miR-4732-3p in the context of cuproptosis. Moreover, in HCC cells with AP003469.2 overexpression, downstream cuproptosis markers showed significant changes, characterized by elevated HSP70 and SLC31A1 levels accompanied by reduced FDX1 expression. In contrast, silencing AP003469.2 markedly promoted cuproptosis activation. These effects were also partly reversed by miR-4732-3p mimics or inhibitors. The aforementioned findings provide credence to the theory that AP003469.2 inhibits the degradation of LIPT1 in HCC by binding to miR-4732-3p, which inhibits cuproptosis and encourages tumor growth, migration, and invasion. These results illuminate a novel regulatory mechanism in HCC progression, emphasizing the therapeutic potential of disrupting this axis to sensitize tumors to cuproptosis inducers and improve the patient’s prognosis.

Our study provides strong evidence that lncRNA AP003469.2 has an oncogenic function in HCC, showing its upregulation in tumors and link to poor prognosis. Integrating TCGA analysis, clinical validation, *in vitro* assays, and xenograft models, we uncovered a novel AP003469.2/miR-4732-3p/LIPT1 ceRNA axis that suppresses cuproptosis and drives proliferation, migration, and invasion. These insights position AP003469.2 as a diagnostic biomarker and independent prognostic factor, with therapeutic potential via copper homeostasis modulation supported by mechanistic data like subcellular localization, dual-luciferase assays, and regulation of cuproptosis proteins. From a translational perspective, strategies such as antisense oligonucleotides(ASO-) or siRNA-mediated silencing of AP003469.2 could restore miR-4732-3p activity and enhance cuproptosis sensitivity in HCC. However, challenges including delivery efficiency, off-target effects, and systemic copper toxicity must be addressed before clinical translation. Combining AP003469.2 inhibition with cuproptosis inducers such as Elesclomol represents a promising avenue for future investigation.

Notwithstanding its strengths, this study has several limitations that warrant consideration. It should be noted that this study has examined only the role of AP003469.2 in HCC cell lines and xenograft models, without patient-derived organoids or other liver cancer subtypes, but we mitigated this by validating across multiple cell lines and clinical samples via qRT-PCR for broader applicability; we concentrated on only the ceRNA mechanism with miR-4732-3p and LIPT1, potentially missing alternative pathways like protein interactions, though addressed partially through Western blots of cuproptosis proteins and correlations with genes like ATP7A and DLAT; some limitations include the lack of large-scale cohorts for gender-specific effects and *in vivo* cuproptosis induction, compensated by TCGA subgroup analyses and multivariate Cox regression adjusting for confounders; additionally, the results do not imply applicability to other diseases, and we cannot determine the male-specific survival basis from this data, but initial insights were provided via Kaplan-Meier curves and ROC analyses; our results lack multi-omics integration for copper dynamics, yet functional assays like Cu^2+^ measurements and chemosensitivity tests linked mechanisms to phenotypes. Despite its preliminary nature, this study clearly demonstrates the therapeutic potential of targeting the AP003469.2 axis in HCC. However, these problems could be solved by expanding to diverse populations and advanced models like CRISPR systems in future studies. Additionally, the xenograft experiments used a small sample size (n = 3 per group), in accordance with the 3R principles of animal use. Future studies with larger cohorts are warranted to further consolidate the *in vivo* findings.

## Conclusions

In conclusion, this study identifies a novel lncRNA-mediated ceRNA axis that integrates copper-dependent cell death with HCC progression. By revealing that AP003469.2 promotes tumor growth through sequestration of miR-4732-3p and upregulation of LIPT1, our work highlights a new mechanistic link between noncoding RNA biology and metabolic cell death. Future research should expand clinical validation, explore therapeutic targeting strategies, and investigate broader interactions between lncRNAs and cuproptosis in cancer. Together, these efforts may pave the way for novel RNA-based and metabolism-oriented therapies in HCC.

## Data Availability

The original contributions presented in the study are included in the article/**Supplementary Material**. Further inquiries can be directed to the corresponding authors.

## Supplementary material

Supplementary Figure 1 AP003469.2 is predominantly localized in the cytoplasm. **(A)** Subcellular localization prediction of AP003469.2 using lncLocator. **(B)** Schematic representation of the AP003469.2 construct, indicating the CMV promoter and the region targeted by the probe. **(C)** RNA Fluorescence *In Situ* Hybridization (FISH) for AP003469.2 (red fluorescence), DAPI staining for nuclei (blue fluorescence), and merged images, visually confirming the predominant cytoplasmic localization of AP003469.2. (Scale bar: 20 µm).

Supplementary Figure 2 AP003469.2 as a ceRNA for hsa-miR-4732-3p, and its clinical significance and regulatory mechanism. **(A)** TCGA volcano map of miRNAs with differential expression. **(B)** Venn diagram intersecting downregulated and prognosis-favorable miRNAs (HR < 1), yielding three candidates. **(C–E)** Correlations between AP003469.2 and three candidates. **(F)** miR-4732-3p levels in normal versus tumor tissues (TCGA). **(G, H)** miR-4732-3p expression across pathologic stages **(G)** and histologic grades **(H)** in TCGA. **(I)** ROC curve assessing the diagnostic performance of miR-4732-3p. **(J)** Linear regression showing inverse correlation between AP003469.2 and miR-4732-3p in clinical samples. **(K)** miR-4732-3p expression in paired clinical tissues (N = 20). **(L)** Expression of miR-4732-3p in xenograft cancers. **(M)** Expression of miR-4732-3p in HCC Cells. **(N)** miR-4732-3p expression after AP003469.2 knockdown or overexpression in HEP3B. **(O)** AP003469.2 and miR-4732-3p predicted binding sites. **(P)** Minimum-free-energy secondary structure of the AP003469.2–miR-4732-3p binding region (mfe = −24.6 kcal/mol). **(Q)** The dual luciferase reporter test confirms that AP003469.2 directly binds to miR-4732-3p. (***p* < 0.01; ****p* < 0.001; *****p* < 0.0001).

Supplementary Figure 3 Clinical significance of LIPT1 in HCC and its regulation by lncRNA AP003469.2 and miR-4732-3p. **(A)** Sankey plot depicting connections between cuproptosis-related mRNAs and lncRNAs, highlighting AP003469.2 within the network. **(B)** LIPT1 expression comparison in TCGA tumor versus normal tissues. **(C)** LIPT1 levels across pathologic stages in TCGA. **(D)** LIPT1 levels across histologic grades in TCGA. **(E)** ROC analysis evaluating diagnostic performance of LIPT1. **(F)** Kaplan–Meier curves indicating worse overall survival with high LIPT1 expression. **(G)** qRT-PCR showing higher LIPT1 in tumors versus matched normals (N = 20). **(H)** LIPT1 expression in HCC cell lines. **(G)** ROC analysis evaluating diagnostic performance of LIPT1. **(I)** LIPT1 expression in xenograft tumors by qRT-PCR. **(J)** LIPT1 protein expression in xenograft tumors by Western blot. **(K)** Spearman correlation showing a positive association between AP003469.2 and LIPT1 in TCGA. **(L)** Spearman correlation showing an inverse association between miR-4732-3p and LIPT1 in TCGA. **(M)** Linear regression in clinical samples confirming a positive AP003469.2–LIPT1 correlation. **(N)** Linear regression in clinical samples confirming a negative miR-4732-3p–LIPT1 correlation. **(O)** Predicted binding sequence between LIPT1 3′UTR and miR-4732-3p. **(P)** Minimum-free-energy secondary structure of the miR-4732-3p/LIPT1 3′UTR complex (mfe = −16.3 kcal/mol). **(Q)** Dual-luciferase reporter assay demonstrating direct regulation of WT but not mutant LIPT1 3′UTR by miR-4732-3p mimics. (**p* < 0.05, ****p* < 0.001, *****p* < 0.0001.).

Supplementary Figure 4 AP003469.2 regulates LIPT1 expression in HCC cells. **(A)** qRT-PCR measurement of LIPT1 mRNA in HEP3B cells. **(B)** qRT-PCR measurement of LIPT1 mRNA in HEPG2 cells. **(C)** Western blot showing LIPT1 protein levels in HEP3B and HEPG2. **(D)** Quantification of LIPT1 protein in HEP3B derived from **(C)**. **(E)** Quantification of LIPT1 relative protein expression in HEPG2 cells based on **(C)**. (**p* < 0.05, ***p* < 0.01, ****p* < 0.001.).
