# Supplementary material for: LncRNA AP003469.2 promotes hepatocellular carcinoma progression by regulating cuproptosis via the miR-4732-3p/LIPT1 axis
Source: Front Oncol. 2026 Oct 1;16:1874176. doi: 10.3389/fonc.2026.1874176 (PMC13669170; doi:10.3389/fonc.2026.1874176)
Supplement: Supplementary file 1 [file Table1.docx]

Supplementary Table 1 The relative expression levels of 8 cuproptosis-related lncRNAs associated with prognosis in HCC and adjacent tissues

| id | logFC | P-Value | FDR |
| --- | --- | --- | --- |
| AC107959.3 | 2.076797 | 3.11E-10 | 5.62E-09 |
| CAPN10-DT | 2.165114 | 3.03E-39 | 4.45E-36 |
| AL606489.1 | 2.965842 | 8.26E-27 | 2.83E-24 |
| SNHG4 | 2.603191 | 5.18E-26 | 1.52E-23 |
| AC007639.1 | 3.251508 | 1.41E-13 | 4.70E-12 |
| AC011921.1 | 2.255455 | 1.30E-24 | 2.99E-22 |
| AC091057.1 | 2.182402 | 1.10E-21 | 1.53E-19 |
| **AP003469.2** | **3.846538** | **1.41E-14** | **5.53E-13** |

Note: logFC (Log Fold Change, logarithmic fold change) indicates the size and direction of gene expression differences between tumor and normal tissues, with positive values indicating higher expression in tumor tissues and negative values indicating lower expression. FDR (False Discovery Rate, false discovery rate) controls the proportion of false positives in multiple hypothesis testing, and a lower FDR value indicates more reliable results.
