# Supplementary material for: LncRNA AP003469.2 promotes hepatocellular carcinoma progression by regulating cuproptosis via the miR-4732-3p/LIPT1 axis
Source: Front Oncol. 2026 Oct 1;16:1874176. doi: 10.3389/fonc.2026.1874176 (PMC13669170; doi:10.3389/fonc.2026.1874176)
Supplement: Supplementary file 2 [file Table2.docx]

Supplementary Table 2 Results of univariate and multivariate COX regression analyses of AP003469.2

| Characteristics | Total(N) | HR(95% CI) Univariate analysis | P value Univariate analysis | HR(95% CI) Multivariate analysis | P value Multivariate analysis |
| --- | --- | --- | --- | --- | --- |
| AP003469.2 | 373 |  |  |  |  |
| Low | 187 | Reference |  | Reference |  |
| High | 186 | 1.735 (1.223 - 2.463) | 0.002 | 1.735 (1.223 - 2.463) | 0.002 |

Note: P-value threshold: 0.1, adjusting factors include gender, age, pathological stage, and histological stage.
